# Supplementary material for: Free Methylglyoxal and Lactate Produced and Released by Cultured Cancer and Non-Cancer Cells: Implications for Tumor Growth and Development
Source: Cells. 2025 Jun 19;14(12):931. doi: 10.3390/cells14120931 (PMC12190829; doi:10.3390/cells14120931)
Supplement: Supplementary file 1 [file cells-14-00931-s001.zip › cells-3646835-supplementary.pdf]

Supplementary materials

Table S1 summarizes our data, showing the significant increase in free MG levels by most cancer cell lines cultured in normoxic conditions and glucose-enriched medium.

Table S1. Free MG release by cancer cell lines cultured in normoxic low or high glucose concentration.

Cancer cell lines	Tissue origin	Mean free MG release +/-SD (nmol/10^6cells)		p*
		Low glucose	High glucose	
MDA-MB-231	Breast	158.9+/-11.33	352.4+/-33.59	<0.0001
MDA-MB468	Breast	144.1+/-3.18	267.4+/-19.73	<0.0001
MCF-7	Breast	242.9+/-27.39	200.1+/-15.97	0.18
U251	Brain	209.1+/-15.02	396.4+/-28.38	<0.0001
U87	Brain	175.1+/-12.46	768.1+/-101.92	<0.0001
PC3	Prostate	253.0+/-7.80	636.9+/-62.92	<0.0001
A549	Lung	156.6+/-34.34	396.3+/-45.85	<0.0001
H1299	Lung	143.5+/-3.93	364.1+/-110.39	<0.0001
BXPC3	Pancreas	227.5+/-31.68	213.9+/-19.68	0.32
HCT116	Colon	184.84+/-8.85	275.70+/-58.14	0.001

p: probability that difference is due to random variation. *Comparison between low and high glucose concentration (α = 0.05).

Table S2 summarizes our data, showing the lake of significant increase in free MG production and release by normal cell lines, except for the NHO osteoblast-derived cell line.

Table S2. Free MG release by normal cell lines cultured in normoxic low or high glucose concentration.

Normal cell lines	Tissue origin	Mean Free MG release +/-SD (pmol/10^6cells)		p*
		Low glucose	High glucose	
NDF	Dermal fibroblasts	790.3+/-54.64	761.7+/-113.8	0.56
NHO	Osteoblasts	1213.0+/-90.21	1530.0+/-62.96	<0.0001
MCF-10A	Epithelial breast cells	437.7+/-65.90	471.8+/-98.65	0.47
SF5	Cutaneous fibroblasts	1135.0+/-234	1389.0+/-184.8	0.17
HUVEC	Umbilical cord-derived endothelial cells	975.2+/-39.35	1104.0+/-153.6	0.23

p: probability that difference is due to random variation. *Comparison between low and high glucose concentration (α = 0.05).

Table S3 summarizes our data showing the free MG and lactate release by non-transformed normal fibroblasts cultured in either normoxic or hypoxic conditions in low or high glucose concentration.

Table S3. Free MG and lactate release by PCS 201-012 normal fibroblasts cultured in normoxic or hypoxic conditions.

	Normoxy		Hypoxy				
	Low glucose	High glucose	Low glucose	High glucose	p*	p**	p***
Free MG (nmol/10^6 cells)	229.11 +/- 15.10	370.89 +/- 25.31	252.59 +/- 17.09	354.32 +/- 23.68	0.3184	0.6391	0.002
Lactate (mmol/10^6 cells)	19.70 +/- 1.05	19.90 +/- 1.51	22.75 +/- 0.88	20.30 +/- 1.32	0.0412	0.8563	0.756

p: probability that difference is due to random variation. * Comparison between normoxic or hypoxic condition in low glucose concentration (α = 0.05). ** Comparison between normoxic or hypoxic condition in high glucose concentration (α = 0.05). *** Comparison between low or high glucose concentration in normoxic condition (α = 0.05).

Table S4 summarizes our data showing comparison between free MG release by non-transformed fibroblasts and fibroblastic cells lines

Table S4. Free MG release by normal fibroblasts compared to normal fibroblastic cell lines in normoxic low or high glucose conditions.

Fibroblastic cells	Mean Free MG release +/-SD (nmol/10^{6}cells)		*p**	*p***	*p****
	**Low glucose**	**High glucose**			
HDF-DJO	95.68 +/-24.45	163.54+/-36.44	<0.001	ND	ND-
PCS 201-012	229.11 +/- 15.10	370.89 +/- 15.31	0.002	-	-
NDF	790.3 +/- 54.64	731.7 +/- 113.8	0.56	<0.0001	<0.0001
SF5	1034 +/- 234	1469 +/- 184.8	0.17	<0.0001	<0.0001

ND: Not determined. *p*: Probability that difference is due to random variation. *Comparison between low and high glucose concentration (α = 0.05).** Comparison between levels of free MG release in the culture medium from PCS 201-012 with those of NDF or SF5 cell lines in normoxic low glucose concentration (α = 0.025). *** Comparison between free MG release in the culture medium from PCS 201-012 with those of NDF or SF5 cell lines in normoxic high glucose concentration (α = 0.025).

Table S5 summarizes our data showing comparison between free MG release by non-transformed fibroblasts and cancer cell lines in normoxic or hypoxic conditions in low or high glucose concentration.

Table S5. Free MG release by normal fibroblasts and cancer cell lines cultured in normoxic or hypoxic conditions.

	Normoxy		Hypoxy				
	**Low glucose**	**High glucose**	**Low glucose**	**High glucose**	*p**	*p***	*p****
PCS 201-012)	229.11 +/- 15.10	370.89 +/- 25.31	252.59 +/- 17.09	354.32 +/- 23.68	0.32	0.64	-
U251	100.25 +/- 8.68	227.79 +/- 26.52	112.26 +/- 9.44	206.35 +/- 13.58	0.36	0.46	0.0012
U87	209.19 +/- 12.52	2655.27 +/- 286.83	232.23 +/- 12.45	2301.49 +/- 258.98	0.21	0.37	<0.0001
HL-60	679.57 +/- 65.62	1316.29 +/- 114.04	818.41 +/-226.46	1696.64 +/- 213.77	0.16	0.10	0.0004

p: probability that difference is due to random variation. * Comparison between normoxic and hypoxic conditions in low glucose concentration (α = 0.05). ** Comparison between normoxic and hypoxic conditions in high glucose concentrations (α = 0.05). *** Comparison between free MG release levels in the culture medium from PCS 201-012 normal fibroblasts with free MG levels from U251, HL60 and U87 cancer cell lines in normoxy high glucose concentrations (α = 0.05/3=0.016).

Table S6 summarizes our data showing comparison between lactate release from normal cell lines and from cancer cell lines in normoxic low or high glucose concentration.

Table S6. Lactate release by normal and cancer cell lines in normoxic low or high glucose concentration.

Cell lines	Mean lactate +/-SD (mmol/10^{6}cells)		*p**	*p***
	**Low glucose**	**High glucose**		
MCF-10A	1.66 +/- 0.01	0.99 +/- 0.01	<0.0001	-
SF5	10.33 +/- 0.99	13.00 +/- 1.33	0.04	-
HUVEC	2.33 +/- 0.01	6.33 +/- 0.67	<0.0001	-
U87	23.10 +/- 1.36	40.27 +/- 4.49	0.002	<0.0001
U251	8.38 +/- 0.25	11.99 +/-1.35	0.017	<0.0001

HL-60	41.84 +/- 8.19	39.39 +/- 7.76	0.74	<0.0001

p: probability that difference is due to random variation. * Comparison between low and high glucose concentration in normoxic conditions (α = 0.05). ** Comparison between the SF5 normal cell line and the U87, U251 and HL60 cancer cell lines grown in normoxic low or high glucose concentration (α = 0.05/3= 0.016).

Table S7 summarizes our data showing comparison of lactate release by non-transformed fibroblasts and cancer cell lines in normoxic or hypoxic conditions in low or high glucose concentration.

Table S7. Lactate release by cancer and normal cells in normoxic or hypoxic low or high glucose concentrations.

Normal fibroblasts and cancer cell lines	Mean Lactate +/-SD (mmol/10^6cells)						
	normoxic		hypoxic				
	Low glucose	High glucose	Low glucose	High glucose	*p**	*p***	*p****
PCS 201-012	19.70 +/- 1.05	19.90 +/- 1.51	22.75 +/- 0.88	20.30 +/- 1.32	0.04	0.85	-
U87	23.10 +/- 1.36	40.28 +/- 4.49	31.39 +/- 2.86	37.03 +/-3.83	0.019	0.59	<0.0001
U251	8.38 +/- 0.75	11.99 +/-4.03	10.55 +/- 2.22	21.32 +/- 4.14	0.02	0.0004	< 0.0001
HL60	41.84 +/- 8.19	39.39 +/- 7.76	54.59 +/- 16.34	56.13 +/- 11.66	0.16	0.59	< 0.0001

p: probability that difference is due to random variation. * Comparison between the normoxic and hypoxic conditions in low glucose concentration (α = 0.05). **Comparison between the normoxic and hypoxic conditions in high glucose concentration (α = 0.05). ***Comparison with PCS 201-012 in normoxic conditions in low glucose concentration (α = 0.05/3=0.016).

Supplementary method

Methylglyoxal Measurement

Lactate measurement was done according to the method described by Rabbani and Thornalley [40] without the use of [^{13}C3]MG and by replacing trichloroacetic acid by trifluoroacetic acid which does not create interference.

1. Chemicals and technical devices used for methylglyoxal (MG) measurement

All chemicals were analytical grade. Methylglyoxal (MG) (40% aqueous solution), Sodium azide and DETAPAC was supplied by Sigma-Aldrich (St. Louis, MO, USA).. O-phenylenediamine also called 1,2-diaminobenzene (DB) was purchased from AcrosOrganics-Fisher Scientific and 5-methylquinoxaline (5-MQX) from Interchim. Acetonitrile and trifluoroacetic acid (TFA) for High performance liquid chromatography (HPLC) gradient grade were purchased from Carlo Erba and formic acid (99-100%, Normapur) from Grosseron. Water used in these experiments was purified using a Milli-Q Water Purification System (EMD Millipore Corp.). The reversed phase chromatography column (symmetry C18: 3.9x150 mm; 5 µm) and the Symmetry Sentry™ guard column (3.9x20 mm, 5 µm) were purchased from Waters (Milford).

2. Preparation of culture medium

100 µl of culture medium was diluted with 50 µl of TFA (50 µl) and mixed by vortexing. The mixture diluted and mixed by vortexing with 100 µl of Milli-Q water and internal standard (5MQX – 7 picomoles). 175 µl of supernatant obtained by centrifugation (12,000 g, 10 min, 4°C) was supplemented with sodium azide (25 µl) and DB HCl-DETAPAC (50 µl). Each sample were incubated for 4 h in the dark at room temperature.

Derivatized samples were then transferred into an autosampler at 4 °C for analyze. Triplicate free MG measures were done.

We used trifluoroacetic acid (TFA) instead of trichloroacetic acid (TCA) to precipitate proteins because TCA gave us interferences causing background noise.

3. **Preparation of standards**

Calibrating standards containing 0.0625-1.6 μmol of MG in 1 ml of water were prepared. Derivatization was carried out by the procedure described above. Calibration curves were constructed by plotting the pick area ratios of 2-MQX and 5-MQX internal standard against the MG concentrations.

4. **High performance liquid chromatography (HPLC) analysis**

HPLC analysis of 2MQ was performed on an LC-MS 8040 Shimadzu. The column is a Kinetex 2.6 μm EVO C10 100Å size 100 × 2.1 mm column. The sample temperature was maintained at 4 °C in the autosampler. The mobile phase A is 0.1% (vol/vol) Formic acid in water with a linear gradient of 0–100% solvent B over 10 min. The flow rate was 0.2 ml/min. The column was then washed for 5 min with 100% solvent B (0.1% formic acid 50% acetonitrile) and re-equilibrated for 15 min with 100% solvent A; the flow rate was increased to 0.4 ml/min for this stage.

5. **Mass spectrometric detection**

Mass spectrometric detection 2MQ was detected by electrospray positive-ion multiple reaction monitoring (MRM) with a retention time of 6.2 min. The injection volume was 10 μl. Two MRM mass transitions (molecular ion > fragment ion, Da) were recorded for analyte and internal standard. MRM mass transition, collision energy (eV) and cone voltage (V) are as follows: 2MQ—145.1 > 77.1 and 145.1 > 92.1. Other mass spectrometer settings were as follows: capillary voltage, 0.60 kV; extractor voltage, 2.00 V; source temperature, 120 °C; desolvation gas temperature, 350 °C; desolvation gas flow, 900 liters per hour; and cone gas flow, 146 liters per hour.

Lactate Measurement

Lactate measurement was done according to the method described by Tan B et al. [41]. The derivatization was done with o-benzylhydroxylamine (oBHA).

1. **Chemicals and technical devices used for lactate measurement**

All chemicals were analytical grade. Lactic acid-13C3 (LA-13C3), N-(3Dimethylaminopropyl)-N′-ethylcarbodiimide hydrochloride (EDC), o-benzyl hydroxylamine (oBHA) were purchased from Sigma Aldrich (St. Louis, MO, USA). Formic acid (LC/MS grade), acetonitrile, methanol, pyridine, hydrochloric acid and ethyl acetate were purchased from purchased from Carlo Erba. Water used in these experiments was purified using a Milli-Q Water Purification Sys-tem (EMD Millipore Corp.). The reversed phase chromatography column (symmetry C18: 3.9x150 mm; 5 μm) and the Symmetry Sentry™ guard column (3.9x20 mm, 5 μm) were purchased from Waters (Milford).

2. **Preparation of culture medium**

Freshly EDC 1 M was prepared in pyridine buffer (5.4 mL of fuming HCl, 8.6 mL of pyridine and 86 mL of ultrapure water at pH 5.0) and freshly oBHA 1 M solution was prepared in acetonitrile/ultrapure water (3:2). To assure the proper homogeneity of the derivatization reagents, both 1 M EDC and 1 M oBHA solutions were mixed and sonicated. For sample derivatization, 100 μL of the oBHA/EDC mixture was added in the sample vial.

After 60 minutes of reaction at room temperature, 1 mL of ultrapure water was added and the mixture was extracted with 4 mL of ethyl acetate by shaking in a vortex at maximum power during 1 minute. After centrifugation (3800 rpm, 5 minutes), the organic layer was separated and dried under a nitrogen stream in a water bath at 40 ºC and 15 psi. The extracts were reconstituted in 150 μL of ultrapure water/methanol (1/1) resulting in a final 30 fold dilution of the sample. Finally, 10 μL of the mixture was injected into the LC-MS/MS system.

3. **High performance liquid chromatography (HPLC) analysis**

The derivatized samples (10 μl) were injected into an LC–MS Shimadzu 8045. samples were separated by HPLC using a Kinetex C18 Column (2.6μm EVO C18 100Å size 100x2.1 mm) with a two-solvent system (A: water with 0.1% formic acid; B: methanol) and a flow rate of 0.6 ml min-1. The gradient program was as follows: 0 to 1.0 min, 0 to 30% B;

1.0 to 5.5 min, 30 to 85% B; 5.5 to 5.6 min, 85 to 98% B; 5.6 to 6.5 min, 98% B; 6.5 to 8.0 min, 0% B.

4. Mass spectrometric detection

The MS acquisition methods used positive electrospray ionization (ESI) and analysis in multiple reaction monitoring (MRM) mode. The injection volume was 10 µl. Lactate values were expressed in mmol by 106 cultured cells. MRM mass transition, voltage potential and collision energy (CE) are as follows: 196 > 91.2 (CE=25), 196 > 124 (CE =15) and 196 > 65.2 (CE=40) for a retention time of 6.71. In this study, we did not distinguish between the two D and L lactate isomers.
